# Pathways to anhedonia in unmedicated major depressive disorder: the roles of insecure attachment and emotion dysregulation

**DOI:** 10.3389/fpsyt.2026.1883292

**Published:** 2026-07-31

**Authors:** Turceun İleri Akdoğan, Rabia Nazik Ekinci, Hasan Kaya, Erol Göka

**Affiliations:** 1 Department of Psychiatry, Rize State Hospital, Rize, Türkiye; 2 Private Practitioner, Ankara, Türkiye; 3 Department of Psychiatry, Ankara Bilkent City Hospital, Ankara, Türkiye

**Keywords:** anhedonia, attachment, emotion regulation, major depressive disorder, path analysis, unmedicated major depressive disorder

## Abstract

**Background:**

Anhedonia, emotion regulation difficulties, and insecure attachment are clinically relevant features of major depressive disorder (MDD); however, their interrelationships have rarely been examined within a single model in unmedicated clinical samples. We tested whether insecure attachment is associated with depression severity through limited access to emotion regulation strategies and anhedonia.

**Methods:**

The sample comprised 102 medication-free patients with MDD (≥6 months without psychotropic medication) and 102 controls selected using predefined matching criteria. Diagnoses were established using the Structured Clinical Interview for DSM-5 Disorders—Clinician Version. Depression severity and anhedonia were assessed using the clinician-administered Hamilton Depression Rating Scale and Snaith–Hamilton Pleasure Scale—Clinician-Administered Version, respectively; attachment and emotion regulation difficulties were assessed using the self-report Relationship Scales Questionnaire and Difficulties in Emotion Regulation Scale. Group comparisons, multivariable logistic regression, and a path model within the MDD group were conducted, with indirect effects tested using bias-corrected bootstrap resampling.

**Results:**

Patients with MDD showed lower secure attachment, higher fearful and preoccupied/anxious attachment, greater overall emotion regulation difficulties, and higher anhedonia than controls. In multivariable logistic regression, DERS-Strategies (OR = 1.21, 95% CI = 1.07–1.38) and SHAPS-C (OR = 1.54, 95% CI = 1.20–1.99) were independently associated with MDD status (Nagelkerke R² = 0.77). In the path model, fearful (β = 0.377) and preoccupied/anxious (β = 0.220) attachment were associated with DERS-Strategies, which in turn was associated with both SHAPS-C (β = 0.431) and HAM-D (β = 0.271); SHAPS-C was independently associated with HAM-D (β = 0.238). The sequential indirect path from fearful attachment to HAM-D through DERS-Strategies and SHAPS-C was significant (β = 0.163, 95% bootstrap CI = 0.050–0.411, p = 0.007). In exploratory analyses, SHAPS-C was positively associated with MDD status but inversely associated with recurrent episode status (OR = 0.68, 95% CI = 0.53–0.88).

**Conclusion:**

In an unmedicated MDD sample, insecure attachment, limited access to emotion regulation strategies, anhedonia, and depression severity were linked in a coherent sequential association pattern, with DERS-Strategies occupying a central position. The differential association of anhedonia across MDD status and recurrent episode presentation is exploratory and warrants confirmation in longitudinal studies.

## 1 Introduction

Major depressive disorder (MDD) is a common psychiatric disorder affecting millions of individuals worldwide and imposes a substantial burden because of its tendency toward chronicity and treatment resistance (1, 2). Anhedonia is one of the core symptoms of MDD and is clinically important because it is associated with functional impairment, poorer treatment response, and increased suicide risk (3, 4). Contemporary models increasingly conceptualize MDD not merely as a syndrome of mood symptoms but as a heterogeneous disorder involving disturbances in core neurocognitive systems, including reward processing and emotion regulation (5–8). Investigating these mechanisms in unmedicated samples has particular clinical and methodological value, given the complex and sometimes paradoxical effects of antidepressant treatment on anhedonia and reward processing (9).

Anhedonia, commonly defined as a loss of pleasure or interest, is increasingly conceptualized not merely as a symptom of MDD but as a transdiagnostic construct associated with treatment resistance, a more severe clinical course, and increased suicide risk (1, 10). However, the view of anhedonia as a unitary and homogeneous construct has been increasingly challenged by contemporary conceptual models; neurobiological and behavioral evidence suggests that anhedonia comprises distinct components (11, 12). These components may show different patterns across clinical stages of depression, particularly between first-episode and recurrent presentations, although the available empirical evidence remains inconsistent (13–15). In the present study, anhedonia was assessed using the clinician-administered SHAPS-C, which primarily captures consummatory hedonic capacity; this measurement focus should be considered when interpreting the findings.

Difficulties in emotion regulation represent a transdiagnostic process closely associated with MDD (16–18). This construct encompasses not only the intensity of negative affect but also the quality and effectiveness of the strategies individuals use to manage such affect. Meta-analytic evidence indicates that maladaptive strategies, such as rumination and suppression, are positively associated with depressive symptoms, whereas adaptive strategies, such as cognitive reappraisal, are associated with lower levels of depressive symptoms (19). In this context, limited access to adaptive emotion regulation strategies has emerged as one of the most consistent correlates of depressive symptomatology in clinical samples (20). Recent studies further suggest that emotion regulation difficulties may also be linked to anhedonia, particularly through deficits in strategies aimed at enhancing positive affect (21–23). From a developmental perspective, the maturation of emotion regulation capacity is closely related to early attachment experiences (24). Accordingly, the association between insecure attachment styles and depressive symptoms may be partly explained through difficulties in emotion regulation (25–27).

Despite this growing body of literature, three important gaps remain. First, an integrated sequential association linking insecure attachment, emotion regulation difficulties, anhedonia, and depression severity has been examined only to a limited extent within a single model in clinical MDD samples; most existing studies have focused on pairwise associations among these constructs. Second, a substantial proportion of the literature is based on medicated samples. Given the complex effects of antidepressant treatment on anhedonia and reward processing, this may complicate the interpretation of these associations independently of medication-related effects (9). Third, it remains unclear whether anhedonia in MDD represents a homogeneous symptom or a construct that differs across clinical stages, particularly between first-episode and recurrent presentations. Emerging evidence suggests that anhedonia may be a dynamic phenomenon that varies across clinical presentations (8, 28). In addition, the associations between insecure attachment and depressive symptoms have been studied predominantly in Western samples, whereas non-Western clinical contexts remain relatively underrepresented (25).

In light of these gaps, the present study examined three research questions in an unmedicated clinical sample of patients with MDD using a structured diagnostic interview and clinician-administered outcome measures: (1) Do insecure attachment styles show a sequential indirect association with depression severity through emotion regulation difficulties and anhedonia? (2) Is anhedonia independently associated with depression severity beyond this sequential pathway? and (3) Do levels of anhedonia and its clinical correlates differ between first-episode and recurrent MDD presentations? By addressing these questions in a Turkish clinical sample, the study also aimed to provide preliminary evidence regarding the conceptual generalizability of attachment-based models of psychopathology in a non-Western clinical context.

## 2 Methods

### 2.1 Participants and procedure

The study protocol was approved by Ankara City Hospital No. 1 Clinical Research Ethics Committee, E.Kurul-E1-23-3215. All procedures were conducted in accordance with the ethical standards of the relevant ethics committee and with the 1964 Declaration of Helsinki and its later amendments. Written informed consent was obtained from all participants before enrollment.

Between January and June 2023, consecutive patients presenting to the psychiatry outpatient clinic of a tertiary academic medical center in Ankara, Türkiye, were screened for eligibility. During this period, 111 patients with clinically suspected MDD who met the initial eligibility criteria underwent detailed assessment. Of these, 5 declined to participate, 2 were excluded because they exceeded the upper age limit of 65 years, and 2 were excluded because of uncontrolled diabetes mellitus. The final MDD sample therefore consisted of 102 patients. During the same period, one healthy control participant was selected for each patient with MDD from hospital personnel using predefined criteria of the same sex, age within ±5 years, and comparable educational level. The final sample included 102 patients with MDD and 102 control participants, yielding a total sample of 204 participants.

Diagnoses were established by a psychiatrist with 5 years of clinical experience in mood disorders using the Structured Clinical Interview for DSM-5 Disorders—Clinician Version (29) and its Turkish adaptation (30). In the control group, all major modules of the SCID-5-CV were administered to confirm the absence of current and lifetime DSM-5 psychiatric diagnoses.

Inclusion criteria for the MDD group were as follows: (i) a current diagnosis of MDD according to DSM-5 criteria; (ii) age between 18 and 65 years; (iii) no use of any psychotropic medication during the 6 months preceding enrollment; and (iv) ability to understand written materials and provide written informed consent.

Exclusion criteria for all participants were as follows: (i) a lifetime history of psychotic disorder or bipolar disorder; (ii) active substance use disorder within the past 12 months; (iii) known neurocognitive disorder; and (iv) severe or unstable medical or neurological conditions that could mimic depressive symptoms or interfere with cognitive or clinical assessment, such as uncontrolled diabetes mellitus, uncontrolled hypothyroidism, epilepsy, or a history of stroke. Medical conditions were assessed through participant self-report and review of available medical records.

Within the MDD group, episode type was assessed using the SCID-5-CV according to DSM-5 criteria. Patients with no previous history of a major depressive episode were classified as having first-episode MDD, whereas those with at least one previous major depressive episode were classified as having recurrent MDD.

### 2.2 Assessment measures

#### 2.2.1 Structured clinical interview for DSM-5 disorders—clinician version

The SCID-5-CV is a semi-structured diagnostic interview used to assess current and lifetime psychiatric disorders according to DSM-5 criteria (29). Its Turkish adaptation has demonstrated good interrater reliability and validity (30). All interviews were conducted by the same psychiatrist with 5 years of clinical experience in mood disorders.

#### 2.2.2 Hamilton depression rating

The HAM-D is a clinician-administered, 17-item scale used to assess the severity of depressive symptoms. Total scores range from 0 to 52, with higher scores indicating more severe depressive symptomatology (31). The Turkish version has demonstrated acceptable reliability and validity, with a reported Cronbach’s α of 0.75 (32). In the present study, the HAM-D was administered by the same clinician in a separate assessment following the SCID-5-CV interview.

#### 2.2.3 Relationship scales questionnaire

The RSQ is a 30-item self-report measure of adult attachment styles based on the four-category model of Bartholomew and Horowitz, assessing secure, fearful, preoccupied/anxious, and dismissing/avoidant attachment dimensions (33). Items are rated on a 7-point Likert scale ranging from 1 = not at all like me to 7 = very much like me. Scores for each attachment style are calculated by averaging the relevant items, with higher scores indicating greater endorsement of the corresponding attachment style. The psychometric properties of the Turkish version have been reported by Sümer and Güngör (34).

#### 2.2.4 Difficulties in emotion regulation scale

The DERS is a 36-item self-report measure developed by Gratz and Roemer to assess difficulties in emotion regulation across six domains: Awareness, Clarity, Nonacceptance, Impulse, Goals, and Strategies (35). Items are rated on a 5-point Likert scale ranging from 1 = almost never to 5 = almost always. Total scores range from 36 to 180, with higher scores indicating greater difficulties in emotion regulation. The Strategies subscale consists of 8 items and assesses the perceived ability to access effective emotion regulation strategies when experiencing negative affect. The Turkish adaptation by Rugancı and Gençöz has demonstrated good psychometric properties (36).

#### 2.2.5 Snaith–Hamilton pleasure scale—clinician-administered version

The SHAPS-C is a 14-item clinician-administered scale used to assess anhedonia (37). Each item presents a standardized scenario assessing the capacity to experience pleasure from specific everyday situations over the past few days, such as taking a warm bath or listening to favorite music. In the present study, items were scored using the original dichotomous method, with preserved pleasure coded as 0 and loss of pleasure coded as 1. Total scores range from 0 to 14, with higher scores indicating greater anhedonia. The SHAPS-C primarily assesses consummatory hedonic capacity and does not directly measure motivational or anticipatory aspects of reward processing. The psychometric properties of the Turkish version have been reported by Gürcan et al. (38).

The self-report measures, including the RSQ and DERS, were administered during the same session following the clinical interviews. All participants completed the questionnaires under researcher supervision.

### 2.3 Statistical analysis

The distribution of continuous variables was assessed using visual inspection, Shapiro–Wilk and Kolmogorov–Smirnov tests, and skewness–kurtosis values. Between-group comparisons were performed using independent-samples t tests or one-way ANOVA when parametric assumptions were met, and Mann–Whitney U or Kruskal–Wallis tests when these assumptions were not met. When Kruskal–Wallis tests indicated significant group differences, Dunn’s *post-hoc* test was applied. Categorical variables were analyzed using chi-square or Fisher’s exact tests, as appropriate. Bonferroni correction was used for *post-hoc* pairwise comparisons when applicable. Effect sizes were reported according to the type of analysis as Cohen’s d, η²/ω², r, or Cramér’s V, together with 95% confidence intervals whenever possible.

Multivariable logistic regression models were constructed using candidate predictors associated with the outcome at p < 0.10 in univariable analyses. Variable selection was performed using the forward likelihood ratio method. Results were presented as adjusted odds ratios (ORs) with 95% confidence intervals.

Missing data were handled using listwise deletion for non-SEM analyses, whereas full information maximum likelihood (FIML) was used for the path analysis. The path analysis was conducted only within the MDD group using maximum likelihood estimation in AMOS version 24.

Model specification was guided by both theoretical considerations and preliminary empirical evidence from the present sample. Fearful and preoccupied/anxious attachment were retained as exogenous predictors because they were significantly elevated in the MDD group, whereas dismissing/avoidant attachment did not differ between groups (**Table 1**) and secure attachment was conceptually framed as a protective construct distinct from insecurity-driven pathways and therefore not modeled as a predictor in the present framework. An initial path model incorporating all six DERS subscales as parallel intermediate variables between attachment and outcomes exhibited poor fit and substantial multicollinearity among subscales. Based on these results and the largest between-group effect size observed for the Strategies subscale (r = 0.75; **Table 1**), the final model retained DERS-Strategies as the intermediate variable.

**Table 1:** Between-group comparisons of attachment, emotion regulation difficulties, and anhedonia scores.

Scale and Subscale	MDD (n = 102), mean (SD)	Control (n = 102), mean (SD)	p-value	Effect size (r)
Attachment (RSQ)				
Secure	3.8 (1.2)	4.4 (1.1)	**<0.001**	-0.31
Fearful	4.8 (1.4)	3.7 (1.4)	**<0.001**	0.44
Preoccupied/anxious	4.4 (1.3)	3.7 (1.1)	**<0.001**	0.27
Dismissing/avoidant	4.5 (1.0)	4.7 (1.0)	0.272	-0.08
Emotion regulation difficulties (DERS)				
Awareness	14.2 (3.4)	12.9 (2.5)	**0.011**	0.18
Clarity	15.6 (4.8)	9.8 (4.0)	**<0.001**	0.58
Nonacceptance	20.2 (7.3)	10.7 (4.4)	**<0.001**	0.64
Impulse control	15.9 (3.7)	11.8 (3.1)	**<0.001**	0.53
Goal-directed behavior	20.2 (4.6)	13.3 (5.0)	**<0.001**	0.60
Strategies	28.6 (8.2)	13.4 (5.8)	**<0.001**	0.75
Total score	121.6 (24.1)	77.0 (18.7)	**<0.001**	0.73
Anhedonia (SHAPS-C)				
Total score	6.4 (3.4)	1.7 (1.8)	**<0.001**	0.68

**Note:** Values are presented as mean (SD) for descriptive comparability. The Mann–Whitney U test was used for between-group comparisons. Effect sizes were calculated as r = Z/√N, where values of 0.1, 0.3, and 0.5 indicate small, medium, and large effects, respectively. The sign of r indicates the direction of the group difference. MDD = major depressive disorder; RSQ = Relationship Scales Questionnaire; DERS = Difficulties in Emotion Regulation Scale; SHAPS-C = Snaith–Hamilton Pleasure Scale–Clinician-Administered Version. Significant p-values are shown in bold.

In the theoretically specified model, fearful and preoccupied/anxious attachment were specified as exogenous variables with direct paths to the DERS-Strategies subscale. Direct paths were specified from DERS-Strategies to both SHAPS-C total score and HAM-D total score, and from SHAPS-C total score to HAM-D total score. The covariance between fearful and preoccupied/anxious attachment was freely estimated. Standardized path coefficients (β) were reported. Model fit was evaluated using χ²/df, the Comparative Fit Index (CFI), Tucker–Lewis Index (TLI), Root Mean Square Error of Approximation (RMSEA, with 90% confidence interval), and Standardized Root Mean Square Residual (SRMR). Indirect effects were tested using 5,000 bias-corrected bootstrap resamples with 95% confidence intervals.

### 2.4 Power analysis

No formal *a priori* power calculation was performed. A *post-hoc* sensitivity analysis indicated that the case-control sample (102 patients with MDD and 102 controls selected using predefined matching criteria; N = 204) was expected to provide sufficient power to detect medium-to-large effects in logistic regression analyses. However, because the path analysis was conducted only within the MDD group (n = 102), the SEM was restricted to a parsimonious model with observed variables. The limited sample size and low degrees of freedom reduced the precision of model-fit evaluation; therefore, fit indices, particularly RMSEA and TLI, were interpreted cautiously.

To evaluate the consistency of the findings across sex and episode-type subgroups, the direction and magnitude of the main associations were examined in exploratory sensitivity analyses. All analyses were two-tailed, the significance level was set at α = 0.05, and analyses were performed using IBM SPSS Statistics version 27 and AMOS version 24.

## 3 Results

### 3.1 Sample characteristics and group differences

The MDD and control groups were comparable in terms of age, sex, and years of education, consistent with the predefined control-selection criteria. The sociodemographic and clinical characteristics of the participants are presented in **Table 2**. The proportion of single participants was significantly higher in the MDD group than in the control group (χ² = 10.15, p = 0.006), whereas the employment rate was lower (χ² = 9.91, p = 0.002). A history of suicide attempt was more frequent in the MDD group than in the control group (χ² = 24.32, p < 0.001). Within the MDD group, the mean age at illness onset was 26.4 ± 10.4 years; 53.9% of patients were classified as having first-episode MDD and 46.1% as having recurrent MDD. The mean HAM-D score was 22.9 ± 6.0.

**Table 2:** Sociodemographic and clinical characteristics of the MDD and control groups

Variable	MDD (n = 102)	Control (n = 102)	p-value
Age, median (Q1–Q3)	30.0 (22.8–40.3)	32.0 (25.0–40.0)	0.180
Sex, n (%)			—
Female	71 (69.6)	71 (69.6)	
Male	31 (30.4)	31 (30.4)	
Years of education, median (Q1–Q3)	12.0 (12.0–16.0)	14.0 (11.8–16.0)	0.384
Marital status, n (%)			**0.006**
Single	52 (51.0)	37 (36.3)	
Married	37 (36.3)	59 (57.8)	
Divorced/widowed	13 (12.7)	6 (5.9)	
Employment status, n (%)			**0.002**
Employed	34 (33.3)	52 (51.0)	
Other	68 (66.7)	50 (49.0)	
History of suicide attempt, n (%)	24 (23.5)	1 (1.0)	**<0.001**
Clinical characteristics of the MDD group			
Age at onset, mean (SD)	26.4 (10.4)	—	—
Number of episodes, mean (SD)	3.4 (1.6)	—	—
Episode type, n (%)		—	—
First episode	55 (53.9)		
Recurrent episode	47 (46.1)		

**Note:** Continuous variables are presented as median (Q1–Q3) or mean (SD), as appropriate. Categorical variables are presented as n (%). Age and years of education were compared using the Mann–Whitney U test. Categorical variables were compared using chi-square or Fisher’s exact tests, as appropriate. Controls were selected in a 1:1 ratio using predefined criteria of the same sex, age within ±5 years, and comparable educational level. Other employment status included unemployed, retired, students, and homemakers. Significant p-values are shown in bold. MDD = major depressive disorder.

### 3.2 Attachment, emotion regulation difficulties, and anhedonia: between-group comparisons

Patients with MDD had significantly lower secure attachment scores and higher fearful and preoccupied/anxious attachment scores than controls, whereas dismissing/avoidant attachment scores did not differ significantly between groups (**Table 1**). Patients with MDD also scored significantly higher than controls on all DERS subscales. The largest effect sizes were observed for Strategies (r = 0.75), Nonacceptance (r = 0.64), and Goal-directed behavior (r = 0.60). The DERS total score was also markedly higher in the MDD group (r = 0.73). SHAPS-C total scores were significantly higher in patients with MDD than in controls (r = 0.68).

### 3.3 Variables associated with MDD status

In the multivariable logistic regression model constructed using the forward likelihood ratio method, DERS-Strategies score (OR = 1.21, 95% CI = 1.07–1.38, p = 0.003) and SHAPS-C total score (OR = 1.54, 95% CI = 1.20–1.99, p = 0.001) were independently associated with MDD status (**Table 3**). Attachment styles were not independently associated with MDD status in the multivariable model. The model showed good classification performance, with a Nagelkerke R² of 0.77, Hosmer–Lemeshow p = 0.600, and an overall classification accuracy of 89.7%.

**Table 3:** Multivariable logistic regression model for factors associated with major depressive disorder status

Variable	B	SE	Wald χ²	p-value	Adjusted OR	95% CI
Attachment styles						
Fearful	-0.99	0.74	1.8	0.225	0.37	0.09 – 1.58
Preoccupied/anxious	-0.45	0.81	0.31	0.602	0.64	0.13 – 3.11
Dismissing/avoidant	-1.43	0.85	2.83	0.093	0.24	0.04 – 1.27
Emotion regulation (DERS)						
Strategies	0.20	0.06	10.83	**0.003**	1.21	1.07 – 1.38
Anhedonia (SHAPS-C)						
SHAPS-C total score	0.43	0.13	11.54	**0.001**	1.54	1.20 – 1.99
Sociodemographic controls						
Marital status						
Married vs Single	-0.49	0.58	0.72	0.396	0.61	0.20 – 1.88
Divorced/Widowed vs Single	0.38	0.71	0.29	0.699	1.54	0.17 – 13.66

**Model fit:** Cox & Snell R² = 0.58; Nagelkerke R² = 0.77; Hosmer–Lemeshow χ² = 6.42, p = 0.600; overall classification accuracy = 89.7%. **Note:** B = regression coefficient; SE = standard error; OR = odds ratio; CI = confidence interval. Secure attachment and single marital status were used as reference categories. The model was built using the forward likelihood ratio method, and variables retained in the final step are shown. Significant p-values are shown in bold.

### 3.4 Path model: sequential indirect association pattern

To examine the sequential associations among attachment, emotion regulation difficulties, anhedonia, and depression severity, a path model was tested within the MDD group only. Direct effects are presented in **Table 4**, and bootstrap-estimated indirect effects are presented in **Table 5**. Fearful attachment (β = 0.377, p < 0.001) and preoccupied/anxious attachment (β = 0.220, p = 0.016) showed significant direct associations with the DERS-Strategies subscale. DERS-Strategies showed significant direct associations with both SHAPS-C total score (β = 0.431, p < 0.001) and HAM-D total score (β = 0.271, p = 0.008). SHAPS-C total score was also significantly associated with HAM-D total score (β = 0.238, p = 0.020). The explained variance was R² = 0.193 for DERS-Strategies, R² = 0.185 for SHAPS-C, and R² = 0.186 for HAM-D.

**Table 4:** Direct effects estimated in the path model within the MDD group

Path	B	β	SE	z	p-value
Fearful Attachment (RSQ) → DERS-Strategies	2.263	0.377	0.551	4.106	**<0.001**
Preoccupied/anxious Attachment (RSQ) → DERS-Strategies	1.421	0.220	0.592	2.403	**0.016**
DERS-Strategies → Anhedonia (SHAPS-C)	0.184	0.431	0.039	4.675	**<0.001**
DERS-Strategies → Depression severity (HAM-D)	0.191	0.271	0.072	2.654	**0.008**
Anhedonia (SHAPS-C) → Depression severity (HAM-D)	0.393	0.238	0.168	2.335	**0.020**

Explained variance: DERS-Strategies, R² = 0.193; SHAPS-C, R² = 0.185; HAM-D-17, R² = 0.186. Note: B = unstandardized coefficient; β = standardized coefficient; SE = standard error; z = test statistic. R² indicates the explained variance of each endogenous variable. All p-values are two-tailed. DERS = Difficulties in Emotion Regulation Scale; SHAPS-C = Snaith–Hamilton Pleasure Scale–Clinician-Administered Version; HAM-D = Hamilton Depression Rating Scale.

**Table 5:** Indirect effects estimated in the path model within the MDD group using bootstrap analysis

Path	B	95% bootstrap CI	p-value
Fearful Attachment → DERS-Strategies → SHAPS-C	0.416	0.153–0.770	**0.001**
Fearful Attachment → DERS-Strategies → HAM-D	0.431	0.138–0.943	**0.001**
Fearful Attachment → DERS-Strategies → SHAPS-C → HAM-D	0.163	0.050–0.411	**0.007**
Preoccupied/anxious attachment→ DERS-Strategies → SHAPS-C	0.261	0.046–0.570	**0.016**
Preoccupied/anxious attachment → DERS-Strategies → HAM-D	0.271	0.037–0.682	**0.017**
Preoccupied/anxious attachment → DERS-Strategies → SHAPS-C → HAM-D	0.103	0.019–0.301	**0.013**
DERS-Strategies → SHAPS-C → HAM-D	0.072	0.023–0.148	**0.010**

**Note:** B = unstandardized indirect effect; CI = confidence interval. Confidence intervals are bias-corrected bootstrap intervals based on 5,000 resamples. DERS = Difficulties in Emotion Regulation Scale; HAM-D = Hamilton Depression Rating Scale; MDD = major depressive disorder; SHAPS-C = Snaith–Hamilton Pleasure Scale—Clinician-Administered Version.

Model fit indices were as follows: χ²(4) = 3.61, χ²/df = 0.90, GFI = 0.985, AGFI = 0.945, CFI = 1.000, TLI = 1.018, RMSEA = 0.000 (90% CI = 0.000–0.147), and SRMR = 0.039.

Bootstrap analyses indicated significant indirect paths from fearful attachment to SHAPS-C through DERS-Strategies (β = 0.416, 95% bootstrap CI = 0.153–0.770, p = 0.001) and from fearful attachment to HAM-D through DERS-Strategies (β = 0.431, 95% bootstrap CI = 0.138–0.943, p = 0.001). The sequential indirect path from fearful attachment to HAM-D through DERS-Strategies and SHAPS-C was also significant (β = 0.163, 95% bootstrap CI = 0.050–0.411, p = 0.007). The corresponding indirect paths originating from preoccupied/anxious attachment were also significant (**Table 5**).

**Figure 1:**
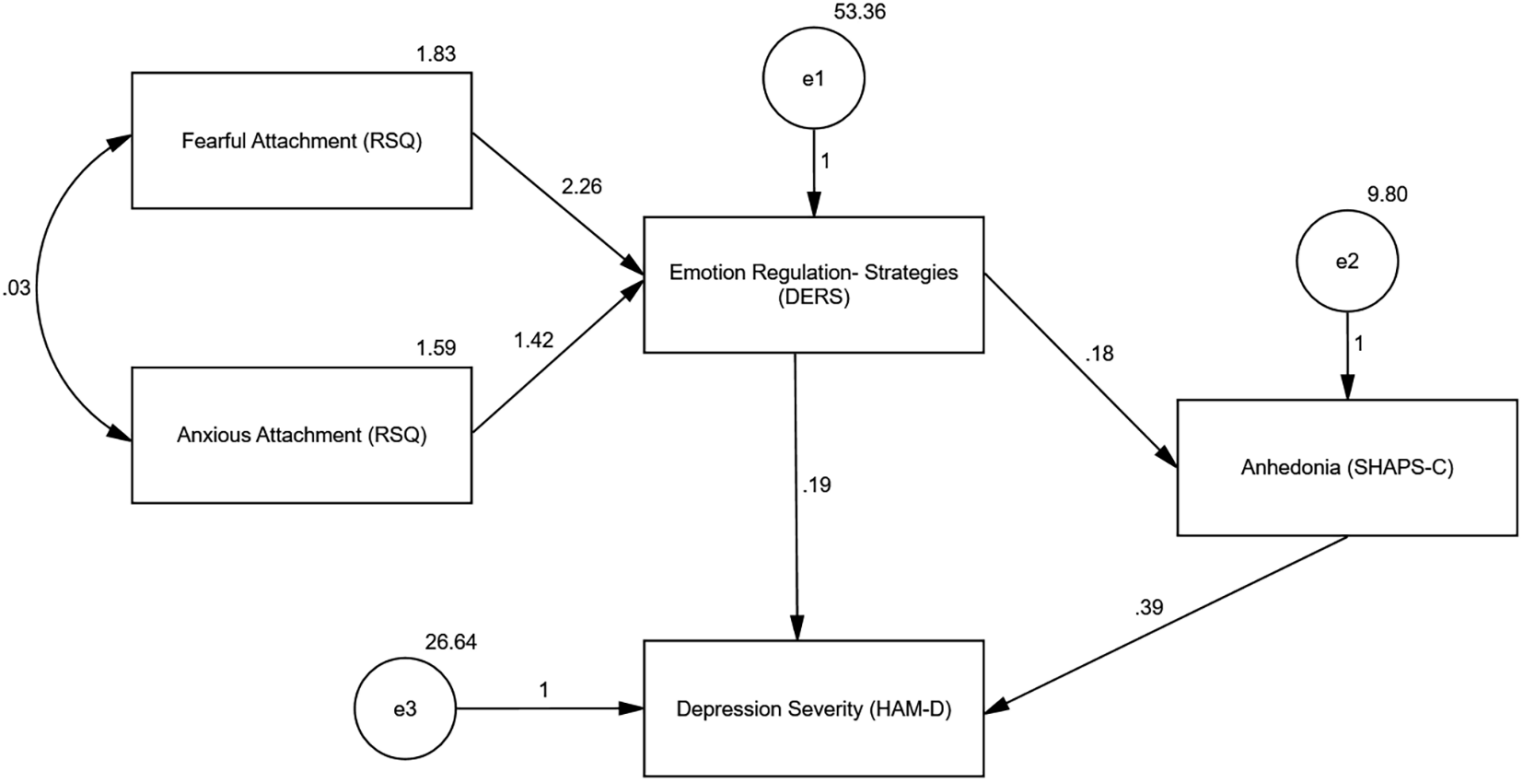
Path model of the sequential associations among insecure attachment, emotion regulation difficulties, anhedonia, and depression severity within the MDD group (n = 102), showing unstandardized coefficients. Fearful and anxious (preoccupied/anxious) attachment (RSQ) are modeled as correlated exogenous variables predicting Emotion Regulation–Strategies (DERS-Strategies), which in turn predicts Anhedonia (SHAPS-C) and Depression Severity (HAM-D); SHAPS-C additionally predicts HAM-D. Circles labeled e1–e3 denote residual (error) variances.

**Figure 2:**
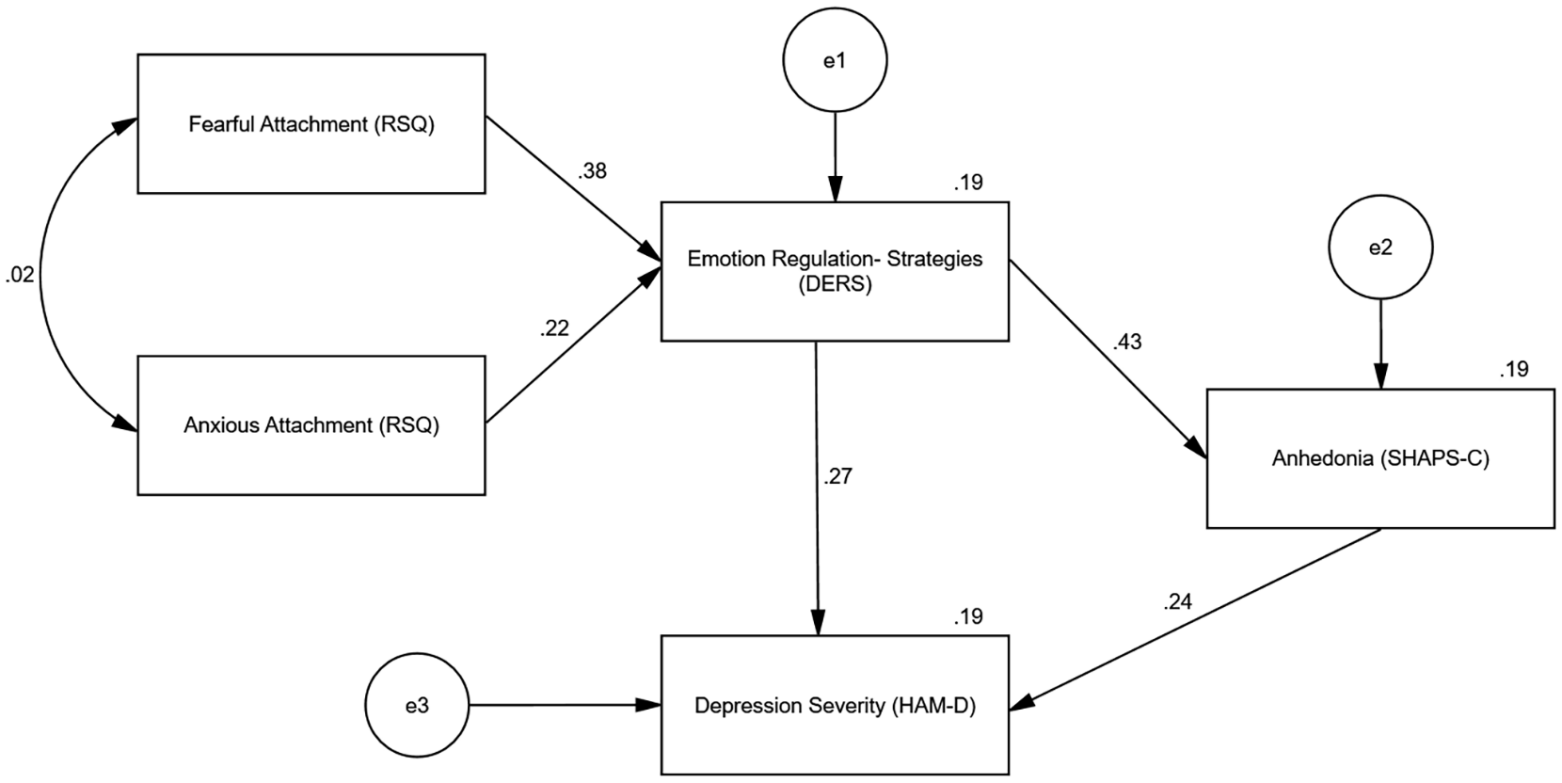
The same path model showing standardized coefficients (β). Fearful and anxious attachment (RSQ) predict Emotion Regulation–Strategies (DERS-Strategies), which predicts Anhedonia (SHAPS-C) and Depression Severity (HAM-D); SHAPS-C additionally predicts HAM-D. Values above the endogenous boxes represent explained variance (R²); circles labeled e1–e3 denote residual variances.

The final path model is shown with unstandardized coefficients in **Figure 1** and standardized coefficients in **Figure 2**.

### 3.5 Exploratory analysis: variables associated with recurrent episode status

A separate multivariable logistic regression analysis was conducted to explore variables associated with recurrent episode status within the MDD group (**Table 6**). Past suicide attempt (OR = 4.75, 95% CI = 1.02–22.14, p = 0.049), DERS Goal-directed behavior score (OR = 1.12, 95% CI = 1.01–1.24, p = 0.030), and educational level were retained in the model. SHAPS-C total score was inversely associated with recurrent episode status (OR = 0.68, 95% CI = 0.53–0.88, p = 0.004). The model had a Nagelkerke R² of 0.52 and an overall classification accuracy of 81.4%.

**Table 6:** Multivariable logistic regression model for factors associated with recurrent episode type among patients with MDD

Variable	B	SE	Wald χ²	p-value	Adjusted OR	95% CI
Clinical history						
Past suicide attempt (yes vs. no)	1.56	0.78	3.99	**0.049**	4.75	1.02 – 22.14
Emotion regulation (DERS)						
Goal-directed behavior	0.11	0.05	4.41	**0.030**	1.12	1.01 – 1.24
Anhedonia (SHAPS-C)						
SHAPS-C total score	-0.38	0.13	8.21	**0.004**	0.68	0.53 – 0.88
Control variables						
Education (high school vs. primary)	-2.48	1.23	4.06	**0.044**	0.08	0.01 – 0.94

Model fit: Cox & Snell R² = 0.39; Nagelkerke R² = 0.52; Hosmer–Lemeshow χ²8 = 4.23, p = 0.836; overall classification accuracy = 81.4%. Note: Analysis was conducted only among patients with MDD n = 102. The dependent variable was episode type, coded as 0 = first episode and 1 = recurrent episode. B = regression coefficient; SE = standard error; OR = odds ratio; CI = confidence interval. Adjusted ORs are shown. The model was built using the forward likelihood ratio method, and variables retained in the final step are displayed. Primary school and no past suicide attempt were used as reference categories. Significant p-values are shown in bold. DERS = Difficulties in Emotion Regulation Scale; MDD = major depressive disorder; SHAPS-C = Snaith–Hamilton Pleasure Scale—Clinician-Administered Version.

To examine whether the inverse association between SHAPS-C and recurrent episode status could be attributed to differences in depression severity, HAM-D scores were compared between first-episode and recurrent MDD groups. HAM-D scores did not differ significantly between the groups (21.94 ± 5.66 vs. 23.21 ± 6.15; Mann–Whitney U = 1118.5, p = 0.304). Detailed scale comparisons by episode type are presented in **Supplementary Table 1**.

### 3.6 Sensitivity analyses

Sensitivity analyses stratified by sex and episode type supported the overall pattern of the primary findings. The associations of DERS-Strategies and SHAPS-C with MDD status, depression severity, and recurrent episode status were directionally consistent across subgroups. Given the limited subgroup sample sizes, these analyses were interpreted as supportive consistency checks rather than confirmatory subgroup tests.

## 4 Discussion

### 4.1 Interpretation of the main findings

This study yielded three main findings in an unmedicated clinical sample of patients with MDD. Its central contribution is that it integrates insecure attachment, limited access to emotion regulation strategies, anhedonia, and depression severity within a single clinically coherent model. First, insecure attachment dimensions showed a sequential association with depression severity through DERS-Strategies and SHAPS-C. Second, DERS-Strategies emerged as a central variable, showing direct associations with both anhedonia and depression severity. Third, in exploratory analyses, SHAPS-C total score was positively associated with MDD status (OR = 1.54) but inversely associated with recurrent episode status (OR = 0.68). These findings are discussed below.

#### 4.1.1 Sequential attachment–emotion regulation–anhedonia–depression pattern

The path model showed that fearful and preoccupied/anxious attachment had significant indirect associations with both anhedonia and depression severity through DERS-Strategies. In addition, the significant sequential indirect paths from fearful and preoccupied/anxious attachment to depression severity through DERS-Strategies and SHAPS-C support an integrated model linking insecure attachment, limited access to emotion regulation strategies, anhedonia, and depression severity. This pattern is conceptually consistent with the sequential structure proposed by attachment-based models of psychopathology (39) and aligns with recent meta-analytic and longitudinal evidence (27, 40).

The stronger association of fearful attachment with DERS-Strategies compared with preoccupied/anxious attachment is noteworthy (β = 0.377 vs. β = 0.220). Although much of the existing literature has emphasized attachment anxiety (27), in the four-category model of Bartholomew and Horowitz (33, 41), fearful attachment is characterized by negative models of both the self and others. This dual negative internal model may be particularly relevant to perceived difficulty accessing effective regulation strategies during emotional distress; the relatively stronger fearful attachment–DERS-Strategies association observed in the present model is consistent with this conceptual framework.

Nevertheless, the observed pattern was derived from a cross-sectional design, and as shown by Maxwell and Cole (42), cross-sectional estimates of indirect effects may be systematically biased when temporal ordering is unknown. Therefore, the present findings should not be interpreted as evidence of causal process, but rather as a cross-sectional association pattern consistent with the proposed sequential ordering. Given the complex effects of antidepressant treatment on anhedonia and reward processing (9), observing this pattern in an unmedicated sample indicates that these associations were examined in a context with fewer medication-related confounding effects.

#### 4.1.2 The central role of DERS-strategies

The second main finding was the central position of the DERS-Strategies subscale in the model. This subscale was independently associated with MDD status in the multivariable logistic regression model (OR = 1.21) and showed significant direct associations with both anhedonia and depression severity in the path model. In addition, among the six DERS subscales, the largest between-group effect was observed for Strategies (r = 0.75). This finding suggests that, in the present sample, one important feature distinguishing the depressive clinical profile may not be only the intensity of negative affect, but also the perceived lack of access to effective strategies for regulating that affect.

This result is consistent with current evidence positioning emotion regulation difficulties as a transdiagnostic process associated with depressive symptomatology (16). Colonnello et al. (20) reported that the association between attachment anxiety and depressive symptoms in a sample of medical students was explained particularly through the Strategies subscale. Ruan et al. (43), using network analysis in a clinical adolescent sample, identified Strategies as one of the central nodes linking depressive and anxiety symptoms. In line with these studies, the present findings suggest that limited access to effective emotion regulation strategies may be linked not only to depression severity but also to anhedonic symptoms.

However, this finding should be interpreted in light of possible conceptual overlap. Some items of the DERS-Strategies subscale, particularly those reflecting beliefs that there is nothing one can do to feel better during emotional distress, may overlap conceptually with helplessness- and hopelessness-related components of depression. Therefore, part of the observed strong associations may reflect genuine emotion regulation difficulties, whereas another part may arise from construct overlap between DERS-Strategies and depressive cognitive content.

#### 4.1.3 The differential association of anhedonia

The third and noteworthy finding was that SHAPS-C total score showed associations in opposite directions with MDD status and recurrent episode status. SHAPS-C scores were strongly and positively associated with MDD status in the case-control model (OR = 1.54, p = 0.001), but were inversely associated with recurrent episode status within the MDD group (OR = 0.68, p = 0.004). The absence of a significant difference in HAM-D scores between first-episode and recurrent MDD groups (p = 0.304) suggests that this pattern is unlikely to be explained solely by differences in depression severity. This finding may indicate a possible episode-type-dependent dissociation between anhedonia and other clinical dimensions of depression. The conceptualization of anhedonia as a homogeneous and linear clinical construct has been increasingly questioned in light of emerging evidence on its subtypes (44, 45), neurobiological correlates (46), and transdiagnostic manifestations (15).

Nevertheless, this finding should be interpreted as hypothesis-generating rather than mechanistic. It is also in the opposite direction to the pattern reported by Ren et al. (47), who found higher anhedonia in recurrent depression among 1,400 first-episode and 487 recurrent depression patients in China. Several explanations may account for this discrepancy. First, medication-free recruitment may have selected against recurrent patients with severe anhedonia, whereas Ren et al.’s recurrent group included patients receiving antidepressants. Second, the SHAPS-C primarily captures consummatory hedonic capacity and does not directly assess motivational or anticipatory anhedonia. In chronic or recurrent depression, consummatory pleasure may be relatively preserved, whereas motivational or anticipatory reward deficits may become more prominent (15, 48). Third, the observed pattern may reflect differences in treatment-seeking behavior or recruitment characteristics rather than the natural course of illness. Therefore, this finding should be regarded as a testable hypothesis regarding whether anhedonia assumes different roles across clinical presentations. Longitudinal studies that follow medicated and unmedicated samples in parallel and separately assess consummatory and motivational/anticipatory anhedonia are needed.

### 4.2 Clinical implications

The findings support three clinical implications. First, skill-based interventions aimed at expanding emotion regulation strategies may represent a clinically meaningful target for both mood and anhedonic symptoms in MDD. This approach may be considered complementary to interventions that directly target positive affect and reward processes (21). Second, the stronger association of fearful attachment with DERS-Strategies compared with preoccupied/anxious attachment suggests that attachment-informed clinical formulations may benefit from addressing both negative self-models and negative other-models in patients with fearful attachment. Third, the possible heterogeneity of anhedonia across clinical presentations suggests that differentiating consummatory and motivational/anticipatory dimensions of anhedonia during clinical assessment may be useful for treatment planning and symptom targeting.

### 4.3 Strengths and limitations

The strengths of this study include the use of an unmedicated clinical MDD sample, which allowed the associations to be examined in a context with fewer medication-related confounding effects; the use of a structured clinical interview for diagnosis and clinician-administered primary outcome measures; and the formal testing of a sequential indirect association pattern linking insecure attachment, limited access to emotion regulation strategies, anhedonia, and depression severity using a path analysis with bootstrap resampling.

The findings should be interpreted in light of several limitations. First, the cross-sectional design precludes causal or temporal interpretation of the observed sequential association pattern. Second, the study was conducted at a single center with a relatively limited sample size; in particular, the first-episode and recurrent MDD subgroups provided limited statistical power for subgroup and interaction analyses. Third, the recruitment of healthy controls from hospital personnel may have introduced healthy-worker and occupational-context selection biases. In addition, although controls were selected in a 1:1 ratio using predefined sex, age, and educational criteria, pair-specific identifiers were not retained; therefore, the matched-pair structure could not be incorporated into the regression analysis using conditional logistic regression. Fourth, the SHAPS-C primarily assesses consummatory hedonic capacity and does not directly measure motivational or anticipatory anhedonia; therefore, the findings cannot be generalized to all dimensions of anhedonia. Emotional blunting was not assessed using a dedicated measure. Although the requirement of at least six months without psychotropic medication reduced the likelihood of blunting related to ongoing antidepressant treatment, residual effects of previous treatment and affective constriction associated with depression itself could not be distinguished from anhedonia. Fifth, the RSQ and DERS were self-report measures, whereas the SHAPS-C and HAM-D were clinician-administered. Although this multimethod approach reduces common-method variance, it does not eliminate possible construct overlap, particularly between DERS-Strategies and helplessness- or hopelessness-related components of depression. Sixth, anxiety symptoms and comorbid anxiety disorders were not systematically assessed. Given their theoretical relevance to attachment, emotion regulation, and anhedonia, their potential contribution to the observed associations could not be evaluated and should be addressed in future studies using standardized anxiety measures. Seventh, the final path model focused on the DERS-Strategies subscale, and alternative models incorporating other DERS subscales were not systematically compared. Finally, although patients were classified by episode type, the precise illness phase at the time of assessment was not determined using a standardized measure; this adds uncertainty to the interpretation of the differential association of anhedonia across episode types. In addition, treatment-resistant depression was not systematically assessed using a predefined operational definition based on the number, adequacy, and duration of previous antidepressant trials. Although none of the included patients had a history of electroconvulsive therapy, the potential influence of previous treatment adequacy and treatment resistance on the observed associations could not be evaluated.

### 4.4 Conclusion

In this unmedicated clinical sample of patients with MDD, the study identified an integrated sequential model linking insecure attachment, limited access to emotion regulation strategies, anhedonia, and depression severity. DERS-Strategies occupied a central position in this pattern, showing direct associations with both anhedonia and depression severity. Fearful attachment showed a stronger association with DERS-Strategies than preoccupied/anxious attachment. The positive association of SHAPS-C total score with MDD status, together with its inverse association with recurrent episode status, represents an exploratory finding suggesting that anhedonia may be heterogeneous across clinical presentations. Confirmation of this pattern requires longitudinal, multicenter studies that separately assess different dimensions of anhedonia and follow medicated and unmedicated samples in parallel.

## Data Availability

The raw data supporting the conclusions of this article will be made available by the authors, without undue reservation.

## Supplementary material

Supplementary Table 1 Comparison of scale scores between first-episode and recurrent MDD groups.
