# Supplementary material for: Pathways to anhedonia in unmedicated major depressive disorder: the roles of insecure attachment and emotion dysregulation
Source: Front Psychiatry. 2026 Jul 31;17:1883292. doi: 10.3389/fpsyt.2026.1883292 (PMC13473420; doi:10.3389/fpsyt.2026.1883292)
Supplement: Supplementary file 1 [file Table1.docx]

| **Supplementary Table S1.** Comparison of clinical and psychological measures between first-episode and recurrent MDD groups | | | | | |  |
| --- | --- | --- | --- | --- | --- | --- |
| **Scale and Subscale** | **First-episode (n = 55) Mean (SD)** | **Recurrent (n = 47) Mean (SD)** | **Test statistic** | **p** | **Effect size (r)** |  |
| **Depression and anhedonia** |  |  |  |  |  |  |
| HAM-D total | 21.94 (5.66) | 23.21 (6.15) | U = 1118.5 | 0.304 | — |  |
| SHAPS-C total | 6.94 (3.76) | 5.74 (2.93) | t = 1.769 | 0.080 | — |  |
| **Attachment (RSQ)** |  |  |  |  |  |  |
| Secure | 3.90 (1.23) | 3.66 (1.14) | U = 1135.5 | 0.362 | — |  |
| Fearful | 4.63 (1.26) | 5.04 (1.47) | U = 974.5 | **0.045** | -0.20 |  |
| Anxious (Preoccupied) | 4.42 (1.27) | 4.37 (1.26) | U = 1247.0 | 0.880 | — |  |
| Avoidant (Dismissive) | 4.42 (0.97) | 4.64 (1.06) | U = 1117.5 | 0.301 | — |  |
| **Emotion regulation (DERS)** |  |  |  |  |  |  |
| Awareness | 14.19 (3.22) | 14.19 (3.63) | U = 1226.5 | 0.771 | — |  |
| Clarity | 15.48 (4.53) | 15.68 (5.00) | t = -0.21 | 0.834 | — |  |
| Non-acceptance | 19.72 (7.66) | 20.68 (6.93) | U = 1170.5 | 0.501 | — |  |
| Impulse control | 15.80 (3.46) | 15.87 (4.11) | U = 1248.5 | 0.889 | — |  |
| Goal-directed behavior | 19.31 (4.83) | 21.17 (4.08) | U = 966.0 | **0.038** | -0.21 |  |
| Strategies | 27.83 (8.50) | 29.47 (7.88) | U = 1140.0 | 0.379 | — |  |
| DERS total | 119.39 (23.13) | 123.68 (25.38) | t = -0.889 | 0.376 | — |  |
| **Note:** Mann–Whitney U test was used for variables with non-normal distributions; independent samples t-test was used for normally distributed variables. Mean (SD) values are presented. Effect size (r) was calculated as Z/√N for Mann–Whitney U comparisons, computed only for statistically significant differences. Significant p values (<0.05) are shown in bold. Abbreviations: HAM-D = Hamilton Depression Rating Scale; SHAPS-C = Snaith–Hamilton Pleasure Scale, Clinician-administered; RSQ = Relationship Scales Questionnaire; DERS = Difficulties in Emotion Regulation Scale. | | | | | |  |
|  |  |  |  |  |  |  |
|  |  |  |  |  |  |  |
|  |  |  |  |  |  |  |
|  |  |  |  |  |  |  |
|  |  |  |  |  |  |  |
